# Relationship between hemoglobin glycation index and mild cognitive impairment risk in middle-aged and elderly people: a cohort study from CHARLS 2011–2018

**DOI:** 10.3389/fneur.2026.1852076

**Published:** 2026-07-14

**Authors:** Dan Guo, Xin Xu

**Affiliations:** Department of Neurology, Beijing Fengtai Hospital of Integrated Traditional Chinese and Western Medicine, Beijing, China

**Keywords:** CHARLS, hemoglobin glycation index, middle-aged and elderly people, mild cognitive impairment, risk

## Abstract

**Background:**

Blood glucose levels may be correlated with cognitive function. The hemoglobin glycation index (HGI) quantifies the difference between observed and predicted glycated hemoglobin (HbA1c) to characterize individual variation in hemoglobin glycation susceptibility. This analysis aimed to explore the association between HGI values and mild cognitive impairment (MCI) risk in middle-aged and elderly people.

**Methods:**

This cohort study included non-MCI participants (aged ≥45 years) from the China Health and Retirement Longitudinal Study (CHARLS) database in 2011, with follow-up data collected through 2018. The association between HGI and MCI risk was investigated by Cox regression analyses, and the results were summarized as hazard ratio with 95% confidence interval [HR (95%CI)]. Further analyses were carried out according to different characteristic subgroups. The restricted cubic splines (RCS) curve was applied to examine the non-linear relationship.

**Results:**

A total of 5,723 participants were included in this analysis, of whom 1,088 (19.01%) had MCI and 4,635 (80.99%) did not. HGI exhibited a significant U-shaped relationship with the risk of MCI (*p* < 0.05). When HGI was classified by quintiles, Q1 [HR = 1.22 (1.00–1.49), *p* = 0.045] and Q5 [HR = 1.26 (1.03–1.53)] of HGI were associated with a higher risk of MCI versus Q2 of HGI. Furthermore, the relationship between low/high HGI values and higher MCI risk was found in subgroups of age <65 years, males, non-diabetes, and cardiovascular diseases (all *p* < 0.05).

**Conclusion:**

Low and high HGI values were related to a higher MCI risk among middle-aged and elderly people, and HGI values may assist in identifying high-risk populations for MCI.

## Introduction

Cognitive impairment, including conditions ranging from mild cognitive decline to dementia, represents a progressive deterioration of cognitive functions such as memory (1). Mild cognitive impairment (MCI) is an intermediate stage between normal aging and dementia (1). The global prevalence of MCI in the elderly population is approximately 23.7%, and more than 50% of MCI patients may develop dementia within 5 years (1, 2). Among the lifetime disease burden related to cognitive impairment, 41% is attributable to MCI (3). The main risk factors for cognitive impairment include advanced age, male sex, diabetes, hypertension, obesity, physical inactivity, depression, and smoking (4–6). Early identification of high-risk populations for MCI and appropriate intervention can help prevent the onset of dementia and reduce the burden associated with dementia.

Hyperglycemia is a common metabolic disorder among older adults and is associated with cognitive decline (7, 8). Glycated hemoglobin (HbA1c) is a commonly used marker that reflects the average blood glucose level over the past 2–3 months, but the HbA1c levels and fasting plasma glucose (FPG) levels are not completely consistent in some populations (9, 10). The average lifespan of red blood cells, differences in the transmembrane glucose gradient of cell membranes, and enzyme abnormalities may affect the level of HbA1c, which makes the measured HbA1c values unable to fully reflect the blood glucose metabolism status (11, 12). Hemoglobin glycation index (HGI) was proposed to quantify the magnitude and direction of the difference between observed and predicted HbA1c in individuals, to characterize individual differences in hemoglobin glycation susceptibility (9). HGI is correlated with the progression of metabolic diseases in both diabetic and non-diabetic populations (13, 14). High HGI may reflect an increase in advanced glycation end products, which are typically linked to inflammation and oxidative stress (15, 16), while low HGI may be caused by stress-induced hyperglycemia (17). High HGI may also be associated with telomere attrition (18), as telomere length is a marker of aging, and telomere attrition can lead to the progression of neurodegenerative diseases (19). A recent cross-sectional study showed that high HGI was related to lower cognitive function in elderly patients with hypertension (20). However, high-quality longitudinal evidence investigating the association between HGI levels and the risk of MCI remains lacking. Thus, this study aimed to explore the correlation between HGI values and MCI risk in middle-aged and elderly people based on large-sample longitudinal data, providing evidence for the identification of high-risk MCI patients and the prevention of dementia.

## Methods

### Study design and populations

The analytical data employed in this cohort study were extracted from the China Health and Retirement Longitudinal Study (CHARLS) database, 2011–2018. CHARLS, a national longitudinal family interview survey, uses a multistage stratified sampling method to evaluate the health status of community residents aged ≥45 years in China.^1^ CHARLS covers 150 regions and 450 villages in 28 provinces in China, collecting information on 17,708 middle-aged and elderly adults. The baseline survey was launched in 2011, followed by four rounds of follow-up surveys in 2013, 2015, 2018 and 2020. Detailed descriptions of the CHARLS design and data collection procedures have been published previously (21). Ethical approval for CHARLS was obtained from Peking University’s Ethics Committee, with written informed consent acquired from all participants. This study initially involved individuals recorded in the 2011 CHARLS survey. Individuals were excluded according to the following criteria: (1) individuals aged <45 years or >89 years, (2) individuals with memory-related disease at baseline (e.g., Alzheimer’s disease, brain atrophy, Parkinson’s disease), (3) individuals with missing data on FPG, (4) individuals without complete data on HbA1c, (5) individuals who provided non-fasting blood samples, (6) individuals diagnosed with MCI at baseline, and (7) individuals without cognition assessment during follow-up.

### Outcomes

The occurrence of MCI during follow-up served as the study outcome. MCI was identified through cognitive function assessments, which comprised episodic memory and mental status. The total cognitive score ranged from 0 to 31, with higher scores reflecting better cognitive performance (22, 23). The CHARLS cognitive function test was adapted from the Telephone Interview for Cognitive Status (TICS), the Mini-Mental State Examination (MMSE), and the Consortium to Establish a Registry for Alzheimer’s disease (CERAD) (24, 25). Previous studies have confirmed that CHARLS cognitive scores are strongly correlated with the Clinical Dementia Rating Scale (CDR) (24). The episodic memory score ranges from 0 to 20 points, consisting of immediate (0–10 points) and delayed (0–10 points) word recall. Participants were asked to immediately recall 10 Chinese words after hearing them, and then recall them again after a 5-min delay. Each correctly recalled word was awarded 1 point.

The mental status score ranges from 0 to 11 and consists of orientation (0–5 points), calculation (0–5 points), and drawing (0–1 points) tests. The orientation test requires participants to identify the current year, month, day of the week, and season, with one point earned for each correct answer. The calculation test involves participants subtracting 7 from 100 five times in succession, with one point awarded for each correct calculation. The drawing test requires participants to accurately redraw the displayed shape (e.g., a pentagon), with one point earned for an accurate drawing.

The aging-associated cognitive decline (AACD) criteria (22, 26) were employed to define MCI, and a cognitive function score < [age standard value - standard deviation (SD)] was considered to have MCI. Individuals were categorized into 5-year age groups, and the mean cognitive function score for each age group was used as the age standard value.

### Calculation of HGI

**Figure 1:**
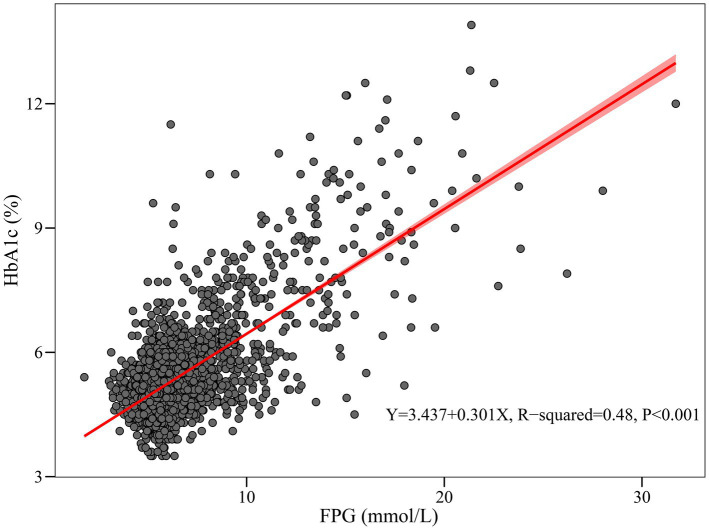
Linear regression analysis of the effect of fasting plasma glucose (FPG) on glycated hemoglobin (HbA1c) levels.

HGI was calculated as: HGI = measured HbA1c - predicted HbA1c (27). A linear regression analysis was performed to model the relationship between FPG and HbA1c in the present cohort for the estimation of predicted HbA1c values (Figure 1). The derived equation was: predicted HbA1c = 0.301 × FPG (mmol/L) + 3.437 (R^2^ = 0.48, *p* < 0.001). In this analysis, HGI was categorized into five groups according to the quintiles (Q1: < −0.368, Q2: −0.368 to −0.132, Q3: −0.132 to 0.068, Q4: 0.068 to 0.322, Q5: ≥ 0.322).

### Data collection

Baseline data on participants were collected, covering age, gender (male, female), education (below high school, high school and above), marital status (married or cohabiting, unmarried), residence (village, town/city), annual income (<10,000, ≥10,000, unknown), smoking (non-current smoking, current smoking), heavy drinking (no, yes), physical activity (insufficient, sufficient, unknown), abdominal obesity (no, yes), C-reactive protein (CRP), hypertension (no, yes), diabetes (no, yes), FBG, HbA1c, dyslipidemia (no, yes), cardiovascular diseases (CVD) (no, yes), emotional problems (no, yes), estimated glomerular filtration rate (eGFR), and follow-up time. The frequency and quantity of alcohol consumption by the participants were converted into the weekly intake of pure alcohol (with one standard drink defined as 14 grams of pure alcohol). Heavy drinking was defined as more than 14.0 standard drinks per week for men and more than 7.0 standard drinks per week for women (28). Diabetes, hypertension, and dyslipidemia were determined according to self-report diagnosis, medication use, and laboratory tests. CVD was identified based on self-report diagnosis. Physical activity was quantified by converting it into weekly metabolic equivalent (MET) minutes corresponding to the intensity of the activity (MET·min/week). Weekly metabolic equivalent = 8.0 × duration of vigorous physical activity + 4.0 × duration of moderate physical activity + 3.3 × duration of low physical activity. Sufficient physical activity was defined as ≥ 600 MET·min/week (29). Based on the CHARLS database, abdominal obesity was defined as a waist circumference of ≥ 90 cm in men and ≥ 85 cm in women (30).

### Statistical analysis

The distributional normality of continuous variables was evaluated utilizing skewness-kurtosis analysis, with homogeneity of variance verified by Levene’s test. Continuous variables were shown as mean ± standard deviation (SD) or median with quartiles [M (Q1, Q3)], and comparisons were conducted utilizing the t-test, t’-test, or Wilcoxon rank-sum test. Categorical variables were reported as count and percentage [*n* (%)], and comparisons were performed using the chi-square test or Fisher’s exact test. Drinking status had the highest missing rate among covariates at 13.56%. The Multiple Interpolation with Chained Equations (MICE) method was used to impute missing values, with a total of 5 iterations generating 5 datasets. The imputation models selected were pmm (Predictive Mean Matching), logreg (Logistic Regression), polr (Proportional Odds Model), and polyreg (Polytomous Logistic Regression) for the imputation, and finally the Rubin rule was chosen to combine the results. The imputation models included the missing situation and the missing variables (education, smoking, gender, heavy drinking, residence, eGFR, and waist circumference). A comparative analysis was carried out to evaluate the differences before and after imputation of missing variables (Supplementary Table 1).

The relationship between HGI and MCI risk was evaluated by the univariate and multivariate Cox regression analyses, and the results were shown as hazard ratio with 95% confidence interval [HR (95%CI)]. Further analyses were carried out according to age, gender, hypertension, diabetes, and CVD subgroups. The multivariate analysis was adjusted for all covariates, including age, gender, education, marital status, residence, annual income, smoking, heavy drinking, physical activity, CRP, hypertension, diabetes, dyslipidemia, CVD, emotional problems, eGFR, and abdominal obesity. The multicollinearity test demonstrated that there was no multicollinearity among these variables included in the multivariate analysis (Supplementary Table 2). The restricted cubic splines (RCS) curve was applied to examine the non-linear relationship between HGI and MCI risk. Statistical analyses were achieved using R 4.5.1 software (Vienna, Austria), with *p* < 0.05 considered significant.

## Results

### Characteristics of participants

**Figure 2:**
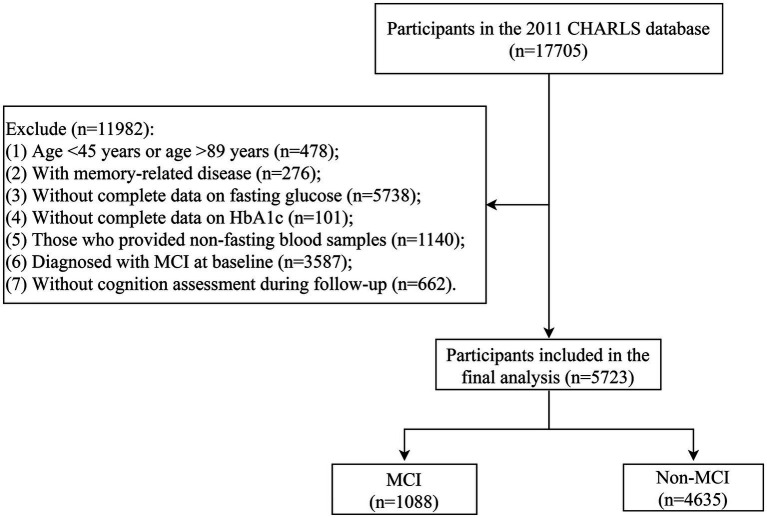
Flowchart of population screening. CHARLS, the China Health and Retirement Longitudinal Study database; MCI, mild cognitive impairment; HbA1c, glycated hemoglobin.

A total of 17,705 participants from the 2011 CHARLS survey were selected for this analysis. After applying exclusion criteria, 11,982 participants were excluded, leaving 5,723 eligible participants for the final analysis (Figure 2). Statistically significant differences in multiple baseline characteristics were observed between included and excluded participants (Supplementary Table 3). The baseline characteristics of included participants are listed in Table 1. Among these 5,723 participants, 1,088 (19.01%) had MCI, while 4,635 (80.99%) did not. The mean follow-up time was 5.44 (±1.97) years. Participants had a mean age of 58.13 (±8.63) years, and 2,777 (48.52%) participants were female. Participants presented a median FBG level of 102.24 (94.68, 112.50) mg/dL, with a median HbA1c level of 5.10% (4.90, 5.40%) and a median HGI value of −0.03 (−0.29, 0.25). Significant differences emerged in gender, education, marital status, residence, annual income, and follow-up time between the MCI and non-MCI groups (*p* < 0.05).

**Table 1:** Characteristics of participants aged ≥45 years.

Variables	Total (*N* = 5,723)	Non-MCI (*N* = 4,635)	MCI (*N* = 1,088)	*p*
Age, years, Mean (±SD)	58.13 (±8.63)	58.08 (±8.72)	58.35 (±8.22)	0.340
Gender, *n* (%)				<0.001
Male	2,946 (51.48)	2,495 (53.83)	451 (41.45)	
Female	2,777 (48.52)	2,140 (46.17)	637 (58.55)	
Education, *n* (%)				<0.001
Below high school	4,852 (84.78)	3,800 (81.98)	1,052 (96.69)	
High school and above	871 (15.22)	835 (18.02)	36 (3.31)	
Marital status, *n* (%)				<0.001
Married or cohabiting	5,197 (90.81)	4,241 (91.50)	956 (87.87)	
Unmarried	526 (9.19)	394 (8.50)	132 (12.13)	
Residence, *n* (%)				<0.001
Village	5,025 (87.80)	4,022 (86.77)	1,003 (92.19)	
Town/city	698 (12.20)	613 (13.23)	85 (7.81)	
Annual income, *n* (%)				<0.001
<10,000	984 (17.19)	798 (17.22)	186 (17.10)	
≥10,000	1,354 (23.66)	1,247 (26.90)	107 (9.83)	
Unknown	3,385 (59.15)	2,590 (55.88)	795 (73.07)	
Smoking, *n* (%)				0.338
No current smoking	3,899 (68.13)	3,144 (67.83)	755 (69.39)	
Current smoking	1824 (31.87)	1,491 (32.17)	333 (30.61)	
Heavy drinking, *n* (%)				0.141
No	5,041 (88.08)	4,068 (87.77)	973 (89.43)	
Yes	682 (11.92)	567 (12.23)	115 (10.57)	
Physical activity, *n* (%)				0.361
Insufficient	418 (7.30)	348 (7.51)	70 (6.43)	
Sufficient	2070 (36.17)	1,683 (36.31)	387 (35.57)	
Unknown	3,235 (56.53)	2,604 (56.18)	631 (58.00)	
Abdominal obesity, *n* (%)				0.371
No	3,242 (56.65)	2,612 (56.35)	630 (57.90)	
Yes	2,481 (43.35)	2023 (43.65)	458 (42.10)	
CRP, M (Q₁, Q₃)	1.05 (0.56, 2.14)	1.06 (0.57, 2.13)	0.99 (0.52, 2.23)	0.160
Hypertension, *n* (%)				0.343
No	3,481 (60.82)	2,805 (60.52)	676 (62.13)	
Yes	2,242 (39.18)	1830 (39.48)	412 (37.87)	
Diabetes, *n* (%)				0.894
No	4,808 (84.01)	3,892 (83.97)	916 (84.19)	
Yes	915 (15.99)	743 (16.03)	172 (15.81)	
FBG, mg/dL, M (Q₁, Q₃)	102.24 (94.68, 112.50)	102.42 (94.86, 112.50)	101.70 (94.32, 112.50)	0.127
HbA1c, %, M (Q₁, Q₃)	5.10 (4.90, 5.40)	5.10 (4.90, 5.40)	5.10 (4.90, 5.40)	0.986
Dyslipidemia, *n* (%)				0.881
No	1880 (32.85)	1,520 (32.79)	360 (33.09)	
Yes	3,843 (67.15)	3,115 (67.21)	728 (66.91)	
CVD, *n* (%)				0.117
No	4,909 (85.78)	3,959 (85.42)	950 (87.32)	
Yes	814 (14.22)	676 (14.58)	138 (12.68)	
Emotional problems, *n* (%)				0.111
No	5,679 (99.23)	4,604 (99.33)	1,075 (98.81)	
Yes	44 (0.77)	31 (0.67)	13 (1.19)	
eGFR, *n* (%)				1.000
<60	108 (1.89)	87 (1.88)	21 (1.93)	
≥60	5,615 (98.11)	4,548 (98.12)	1,067 (98.07)	
Follow time, years, Mean (±SD)	5.44 (±1.97)	5.80 (±1.77)	3.92 (±2.05)	<0.001
HGI, M (Q₁, Q₃)	−0.03 (−0.29, 0.25)	−0.04 (−0.29, 0.25)	−0.01 (−0.30, 0.26)	0.267
HGI, *n* (%)				0.169
−0.368 to −0.132 (Q2)	1,140 (19.92)	953 (20.56)	187 (17.19)	
<−0.368 (Q1)	1,145 (20.01)	921 (19.87)	224 (20.59)	
−0.132 to 0.068 (Q3)	1,148 (20.06)	922 (19.89)	226 (20.77)	
0.068 to 0.322 (Q4)	1,145 (20.01)	923 (19.91)	222 (20.40)	
≥0.322 (Q5)	1,145 (20.01)	916 (19.76)	229 (21.05)	

MCI, mild cognitive impairment; CRP, C-reactive protein; FBG, fasting plasma glucose; HbA1c, glycated hemoglobin; CVD, cardiovascular diseases; eGFR, estimated glomerular filtration rate; HGI, hemoglobin glycation index.

### Association between HGI values and MCI risk in middle-aged and elderly adults

**Figure 3:**
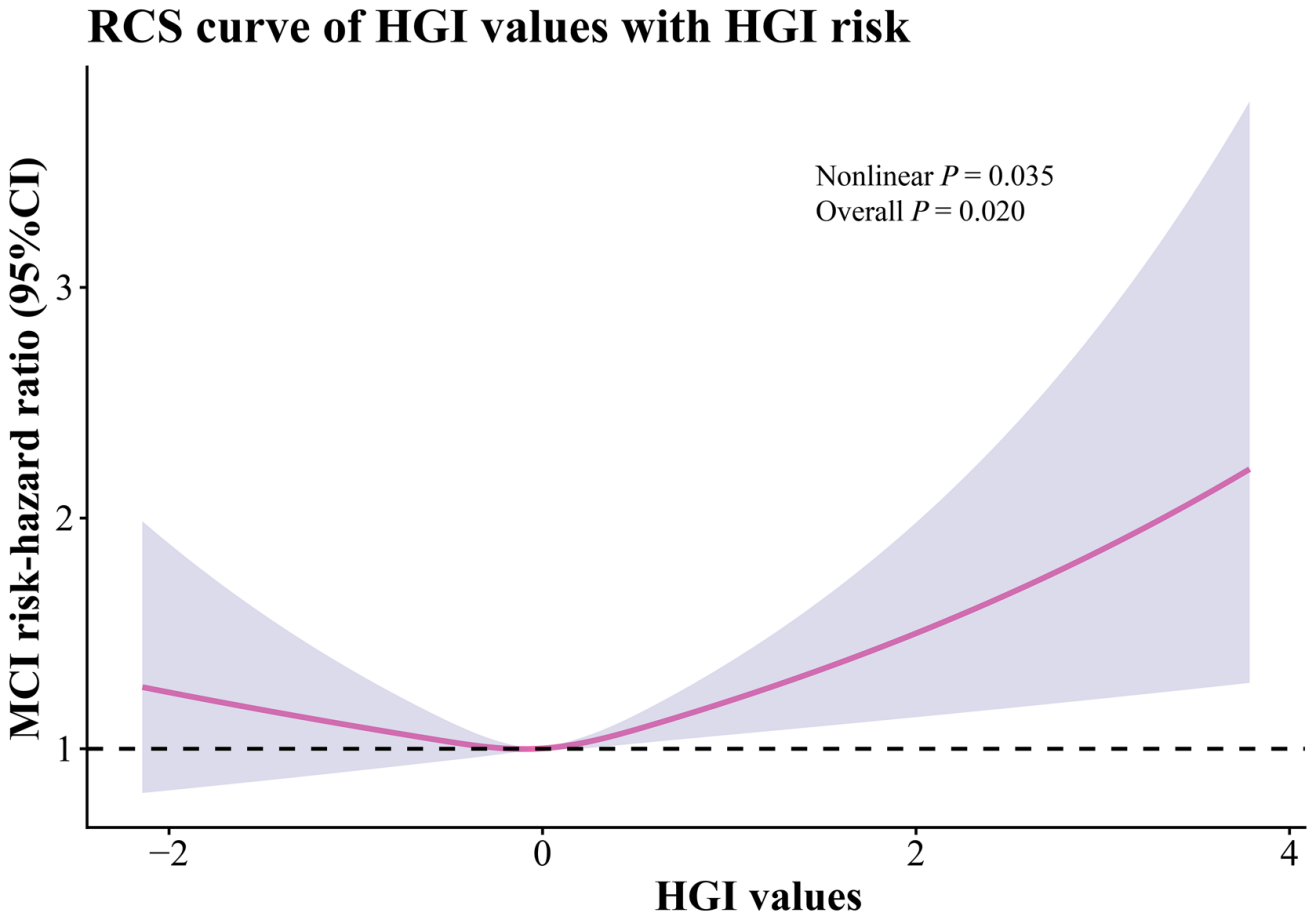
The restricted cubic splines (RCS) curve between HGI values and MCI risk. HGI, Hemoglobin glycation index; MCI, Mild cognitive impairment.

The non-linear relationship between HGI values and MCI risk is listed in Figure 3. There was a significant U-shaped relationship between HGI values and MCI risk (P_overall_ = 0.035, P_nonlinear_ = 0.020). When HGI exceeds a certain value, the MCI risk increases significantly as HGI values rise.

Table 2 presents the Cox analysis results for the relationship between HGI values and MCI risk. For continuous HGI values, univariate Cox analysis found a significant association between HGI values and MCI risk [HR = 1.06 (1.00–1.13), *p* = 0.036], but this association was not observed in multivariate analysis (*p* = 0.075). When HGI was classified by quintiles, Q1 [adjusted HR = 1.22 (1.00–1.49), *p* = 0.045] and Q5 [adjusted HR = 1.26 (1.03–1.53)] of HGI were linked to a higher risk of MCI versus Q2 of HGI in multivariate analysis.

**Table 2:** The Cox analysis results for the relationship between HGI values and MCI risk.

Variables	Univariate		Multivariate	
	HR (95%CI)	*p*	HR (95%CI)	*p*
HGI (continuous)	1.06 (1.00–1.13)	0.036	1.06 (0.99–1.12)	0.075
HGI				
−0.368 to −0.132 (Q2)	Ref		Ref	
<−0.368 (Q1)	1.20 (0.99–1.46)	0.065	1.22 (1.00–1.49)	0.045
−0.132 to 0.068 (Q3)	1.22 (1.01–1.48)	0.044	1.21 (0.99–1.47)	0.057
0.068 to 0.322 (Q4)	1.18 (0.97–1.44)	0.089	1.15 (0.95–1.40)	0.156
≥0.322 (Q5)	1.26 (1.03–1.52)	0.021	1.26 (1.03–1.53)	0.022

MCI, mild cognitive impairment; HGI, hemoglobin glycation index; HR, hazard ratio; CI, confidence interval; Ref, reference; Multivariate Cox regression analysis adjusted for age, gender, education, marital status, residence, annual income, smoking, heavy drinking, physical activity, CRP, hypertension, diabetes, dyslipidemia, CVD, emotional problems, eGFR, and abdominal obesity.

The relationship between HbA1c levels and MCI risk was also explored (Supplementary Table 4). However, no significant association was observed between HbA1c and MCI risk when HbA1c was modeled as a continuous, two-category (<6.5%, ≥6.5%), or three-category variable (<5.7, 5.7 to 6.5%, ≥6.5%) (*p* > 0.05). A comparison of predictive performance between HGI and HbA1c for MCI risk was conducted (Supplementary Table 5). The C-statistic for continuous HGI in predicting MCI risk was slightly higher than that for HbA1c [0.513 (95%CI: 0.494–0.531) vs. 0.502 (95%CI: 0.484–0.520)], without reaching statistical significance (*p* = 0.078). When HGI was modeled as a categorical variable, its C-statistic for MCI risk prediction was significantly greater relative to HbA1c [0.520 (95%CI: 0.502–0.538) vs. 0.501 (95%CI: 0.494–0.508); *p* = 0.033].

**Figure 4:**
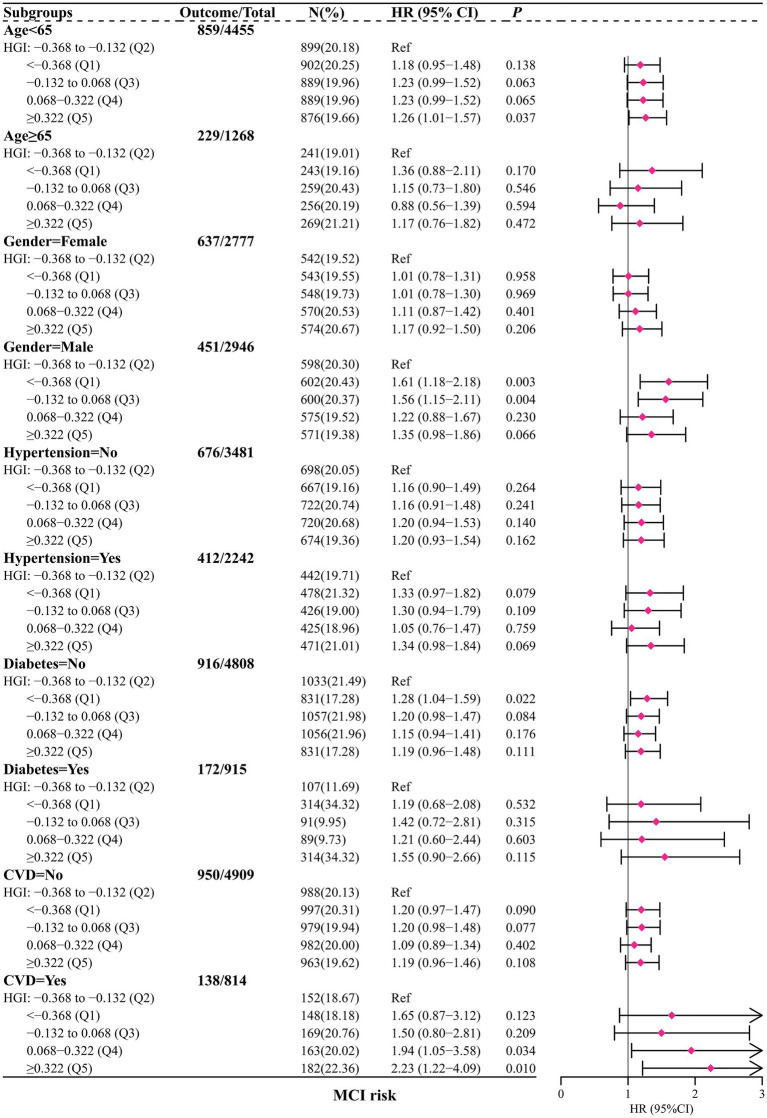
The associations between HGI values and MCI risk in different characteristic subgroups. HGI, Hemoglobin glycation index; MCI, Mild cognitive impairment; CVD, Cardiovascular diseases.

The associations between HGI values and MCI risk in different characteristic subgroups are shown in Figure 4. Low HGI levels were associated with a higher MCI risk in subgroups of males [Q1: HR = 1.61 (1.18–2.18)] and non-diabetes [Q1: HR = 1.28 (1.04–1.59)]. High HGI levels were linked to a higher MCI risk in subgroups of age <65 years [Q5: HR = 1.26 (1.01–1.57)], males [Q3: HR = 1.56 (1.15–2.11)], and CVD [Q4: HR = 1.94 (1.05–3.58); Q5: HR = 2.23 (1.22–4.09)].

## Discussion

This study analyzed the association between HGI values and MCI risk in middle-aged and elderly adults. Our results found a non-linear correlation between HGI values and MCI risk. Low/high HGI values were associated with an elevated MCI risk, and this relationship was found in subgroups of age <65 years, males, non-diabetes, and CVD.

Cognitive impairment has a variety of manifestations, ranging from cognitive decline to debilitating diseases such as dementia (31). Cognitive impairment and subsequent dementia are among the main causes of death in older adults (32). Several studies have reported that blood glucose levels are related to cognitive impairment (8, 33). The duration of diabetes and blood glucose control may influence the type and severity of cognitive impairment (8). Glucose peaks are correlated with cognitive decline and dementia risk in people with diabetes (33). Our analysis examined the correlation between HGI levels and MCI risk in middle-aged and elderly adults using longitudinal data. HbA1c reflects the average blood glucose level over the previous 2–3 months. Nevertheless, discrepancies between HbA1c and FPG are commonly observed across certain populations. HGI quantifies the individual differences between HbA1c and FPG, revealing individual differences in HbA1c susceptibility. Our results indicated that low and high HGI values were associated with an increased MCI risk. High HGI may reflect an increase in advanced glycation end products, which are commonly related to inflammation and oxidative stress (15, 16), while low HGI may be due to high FPG and low HbA1c caused by stress-induced hyperglycemia (17). A cross-sectional study also demonstrated that high HGI was correlated with cognitive decline in hypertensive patients (20). Moreover, compared with persistent hyperglycemia, blood glucose fluctuations may have a greater adverse impact on endothelial function and induce oxidative stress, leading to cognitive decline (34, 35). In clinical practice, it may be necessary to incorporate HGI into a comprehensive assessment system for cognitive impairment, conducting regular cognitive screening and early intervention for both the high and low HGI groups. At the same time, it is important to identify and manage the underlying causes of HGI changes, such as physiological stress and blood glucose fluctuations.

The mechanisms by which high and low HGI affect cognitive impairment are unclear, but may be related to neuronal damage, oxidative stress, inflammation, and insulin resistance (36, 37). Elevated extracellular glucose levels promote the formation of advanced glycation end products, triggering inflammatory responses and oxidative stress in neurons (38). Oxidative stress causes mitochondrial dysfunction and damages various body systems, including the central nervous system (39). Excessive production of reactive oxygen species exceeds endogenous antioxidant capacity, leading to oxidative damage to lipids, proteins, and nucleic acids, as well as the activation of stress-related signaling molecules (20, 40). Free radicals generated during oxidative stress induce chronic inflammation in the brain by releasing pro-inflammatory cytokines, resulting in cellular and synaptic damage, disruption of synaptic function, and activation of microglia, ultimately leading to neuronal damage (41, 42). Hyperglycemia may also cause signal processing dysfunction and neuronal death through direct degradation of myelin, as well as indirectly impairing neurological function by inducing changes in cerebral blood vessels, thereby increasing the incidence of cognitive impairment and dementia (43). Inflammation causes insulin resistance in diabetes, and the disruption of glucose metabolism further increases inflammation (44). Long-term exposure to inflammation is related to the pathogenesis of cognitive impairment and dementia (44). Furthermore, chronic stress in stress-induced hyperglycemia activates the sympathetic nervous system and the hypothalamic–pituitary–adrenal axis, leading to endocrine changes that affect glucose and lipid metabolism as well as insulin sensitivity (37). Insulin plays an important role in regulating learning and memory functions in the brain, and insulin resistance in the brain impairs these functions, leading to poorer cognitive function (45, 46). Although both high and low HGI levels were significantly associated with MCI risk, their underlying pathophysiological mechanisms may differ. Further research is needed to explore the clinical implications of this U-shaped relationship.

This analysis investigated the correlation between HGI and MCI risk in middle-aged and elderly populations through longitudinal data from long-term follow-up. The large sample data with national representativeness ensures the robustness of the results. In addition, subgroup analysis was used to determine the applicability of HGI in populations with specific characteristics. Nevertheless, some limitations should be acknowledged. First, the determination of variables such as comorbidities was obtained through self-reporting, which may be subject to recall bias. Second, although we have adjusted for many potential confounders, there may still be some confounders affecting cognitive function that were not considered (e.g., APOE genotype). Third, the population in this study was from China, and the generalizability of the study results to other populations with different demographic and epidemiological characteristics needs to be further verified. Fourth, during the screening process for the study population, many participants were excluded due to missing key variables (e.g., FPG), which may introduce selection bias. Differences between the participants included in the analysis and those excluded in many characteristics also suggest the possibility of selection bias. Fifth, the HbA1c prediction formula developed in this study is only applicable to the current population. For other populations, a new linear regression model between FPG and HbA1c should be established to derive the corresponding HbA1c prediction formula.

## Conclusion

A significant non-linear association was observed between HGI values and MCI risk among middle-aged and elderly adults. Both low and high HGI values were related to an increased MCI risk, and this correlation persisted across specific subgroups. Future research can construct tools to identify populations at high MCI risk based on HGI values.

## Data Availability

Publicly available datasets were analyzed in this study. This data can be found at: study datasets were obtained from the CHARLS database, https://charls.charlsdata.com/index/zh-cn.html.

## Footnotes

1
https://charls.pku.edu.cn/
