# Supplementary material for: Relationship between hemoglobin glycation index and mild cognitive impairment risk in middle-aged and elderly people: a cohort study from CHARLS 2011–2018
Source: Front Neurol. 2026 Jul 14;17:1852076. doi: 10.3389/fneur.2026.1852076 (PMC13407276; doi:10.3389/fneur.2026.1852076)
Supplement: Supplementary file 1 [file Supplementary_file_1.docx]

**Supplementary Table 1**. The differences before and after imputation of missing variables.

| Variables | Proportion of missing values (n (%)) | Before imputation (N=5723) | After imputation (N=5723) | Statistics | *P* |
| --- | --- | --- | --- | --- | --- |
| Education, n (%) | 1 (0.02%) |  |  | χ² = 0.000 | 1.000 |
| High school below |  | 4851 (84.76) | 4852 (84.78) |  |  |
| High school and above |  | 871 (15.22) | 871 (15.22) |  |  |
| Smoking, n (%) | 1 (0.02%) |  |  | χ² = 0.000 | 1.000 |
| No current smoking |  | 3898 (68.11) | 3899 (68.13) |  |  |
| Current smoking |  | 1824 (31.87) | 1824 (31.87) |  |  |
| Gender, n (%) | 5 (0.09%) |  |  | χ² = 0.000 | 0.991 |
| Male |  | 2945 (51.46) | 2946 (51.48) |  |  |
| Female |  | 2773 (48.45) | 2777 (48.52) |  |  |
| Heavy drinking, n (%) | 776 (13.56%) |  |  | χ² = 0.000 | 1.000 |
| No |  | 5036 (88.00) | 5041 (88.08) |  |  |
| Yes |  | 682 (11.92) | 682 (11.92) |  |  |
| Residence, n (%) | 6 (0.1%) |  |  | χ² = 0.000 | 1.000 |
| Village |  | 5019 (87.70) | 5025 (87.80) |  |  |
| Town/city |  | 698 (12.20) | 698 (12.20) |  |  |
| eGFR, Mean (±SD) | 7 (0.12%) | 96.19 (±14.09) | 96.19 (±14.08) | t = -0.005 | 0.996 |
| Waist circumference, Mean (±SD) | 694 (12.13%) | 85.35 (±12.37) | 85.35 (±11.76) | t' = 0.000 | 1.000 |

Note: eGFR, estimated glomerular filtration rate.

**Supplementary Table 2**. Test for multicollinearity among variables included in the multivariate model.

| Variables | GVIF | Df | GVIF^(1/(2*Df)) |
| --- | --- | --- | --- |
| Age | 1.232 | 1 | 1.11 |
| Gender | 1.806 | 1 | 1.344 |
| Education | 1.031 | 1 | 1.015 |
| Marital status | 1.111 | 1 | 1.054 |
| Residence | 1.028 | 1 | 1.014 |
| Annual Income | 1.104 | 2 | 1.025 |
| Smoking | 1.613 | 1 | 1.27 |
| Heavy drinking | 1.15 | 1 | 1.072 |
| Physical activity | 1.013 | 2 | 1.003 |
| CRP | 1.01 | 1 | 1.005 |
| Hypertension | 1.121 | 1 | 1.059 |
| Diabetes | 1.036 | 1 | 1.018 |
| Dyslipidemia | 1.057 | 1 | 1.028 |
| CVD | 1.05 | 1 | 1.025 |
| Emotional | 1.01 | 1 | 1.005 |
| eGFR | 1.03 | 1 | 1.015 |
| Abdominal obesity | 1.155 | 1 | 1.075 |

Note: GVIF, generalized variance inflation factor; Df, degrees of freedom; CRP, C-reactive protein; CVD, cardiovascular diseases; eGFR, estimated glomerular filtration rate;

Multicollinearity was considered absent when GVIF^(1/(2*Df)) < 5.

**Supplementary Table 3**. Comparison of characteristics between individuals included in the analysis and those excluded.

| Variables | Total (N=17705) | Excluded individuals (N=11982) | Included individuals (N=5723) | *P* |
| --- | --- | --- | --- | --- |
| Follow time, Mean (±SD) | 3.85 (±3.10) | 3.09 (±3.25) | 5.44 (±1.97) | <0.001 |
| Age, Mean (±SD) | 59.05 (±10.15) | 59.49 (±10.78) | 58.13 (±8.63) | <0.001 |
| Gender, n (%) |  |  |  | <0.001 |
| Male | 8471 (47.85) | 5526 (46.12) | 2945 (51.46) |  |
| Female | 9221 (52.08) | 6448 (53.81) | 2773 (48.45) |  |
| Education, n (%) |  |  |  | <0.001 |
| High school below | 15407 (87.02) | 10556 (88.10) | 4851 (84.76) |  |
| High school and above | 2246 (12.69) | 1375 (11.48) | 871 (15.22) |  |
| Marital status, n (%) |  |  |  | <0.001 |
| Married or cohabiting | 15451 (87.27) | 10254 (85.58) | 5197 (90.81) |  |
| No married | 2221 (12.54) | 1695 (14.15) | 526 (9.19) |  |
| Residence, n (%) |  |  |  | 0.103 |
| Village | 15396 (86.96) | 10377 (86.60) | 5019 (87.70) |  |
| Town/city | 2262 (12.78) | 1564 (13.05) | 698 (12.20) |  |
| Annual Income, n (%) |  |  |  | <0.001 |
| <10000 | 3079 (17.39) | 2095 (17.48) | 984 (17.19) |  |
| ≥10000 | 3820 (21.58) | 2466 (20.58) | 1354 (23.66) |  |
| Unknown | 10806 (61.03) | 7421 (61.93) | 3385 (59.15) |  |
| Smoking, n (%) |  |  |  | <0.001 |
| No current smoking | 12034 (67.97) | 8136 (67.90) | 3898 (68.11) |  |
| Current smoking | 4871 (27.51) | 3047 (25.43) | 1824 (31.87) |  |
| Heavy drinking, n (%) |  |  |  | <0.001 |
| No | 15863 (89.60) | 10827 (90.36) | 5036 (88.00) |  |
| Yes | 1675 (9.46) | 993 (8.29) | 682 (11.92) |  |
| Physical activity, n (%) |  |  |  | <0.001 |
| Insufficient | 1314 (7.42) | 896 (7.48) | 418 (7.30) |  |
| Sufficient | 5601 (31.64) | 3531 (29.47) | 2070 (36.17) |  |
| Unknown | 10790 (60.94) | 7555 (63.05) | 3235 (56.53) |  |
| Waist circumference, Mean (±SD) | 84.28 (±12.62) | 83.66 (±12.73) | 85.35 (±12.37) | <0.001 |
| CRP, M (Q₁, Q₃) | 1.04 (0.55, 2.19) | 1.04 (0.54, 2.26) | 1.05 (0.56, 2.14) | 0.793 |
| Hypertension, n (%) |  |  |  | 0.002 |
| No | 11066 (62.50) | 7585 (63.30) | 3481 (60.82) |  |
| Yes | 6639 (37.50) | 4397 (36.70) | 2242 (39.18) |  |
| Diabetes, n (%) |  |  |  | <0.001 |
| No | 15369 (86.81) | 10561 (88.14) | 4808 (84.01) |  |
| Yes | 2336 (13.19) | 1421 (11.86) | 915 (15.99) |  |
| FBG, M (Q₁, Q₃) | 102.42 (94.32, 113.76) | 102.60 (93.96, 115.02) | 102.24 (94.68, 112.50) | 0.468 |
| HbA1c, M (Q₁, Q₃) | 5.10 (4.90, 5.40) | 5.10 (4.80, 5.40) | 5.10 (4.90, 5.40) | 0.074 |
| Dyslipidemia, n (%) |  |  |  | <0.001 |
| No | 9633 (54.41) | 7753 (64.71) | 1880 (32.85) |  |
| Yes | 8072 (45.59) | 4229 (35.29) | 3843 (67.15) |  |
| CVD, n (%) |  |  |  | 0.106 |
| No | 15295 (86.39) | 10386 (86.68) | 4909 (85.78) |  |
| Yes | 2410 (13.61) | 1596 (13.32) | 814 (14.22) |  |
| Emotional, n (%) |  |  |  | <0.001 |
| No | 17454 (98.58) | 11775 (98.27) | 5679 (99.23) |  |
| Yes | 251 (1.42) | 207 (1.73) | 44 (0.77) |  |
| eGFR, Mean (±SD) | 95.66 (±14.64) | 95.14 (±15.13) | 96.19 (±14.09) | <0.001 |

Note: CRP, C-reactive protein; FBG, fasting plasma glucose; HbA1c, glycated hemoglobin; CVD, cardiovascular diseases; eGFR, estimated glomerular filtration rate.

**Supplementary Table 4**. The association between HbA1c and MCI risk.

| Variables | Univariate | | Multivariate | |
| --- | --- | --- | --- | --- |
|  | HR (95%CI) | *P* | HR (95%CI) | *P* |
| HbA1c (continuous) | 1.05 (0.97-1.12) | 0.224 | 1.06 (0.97-1.15) | 0.183 |
| HbA1c |  |  |  |  |
| <6.5% | Ref |  | Ref |  |
| ≥6.5% | 1.05 (0.80-1.38) | 0.726 | 1.13 (0.82-1.57) | 0.442 |
| HbA1c |  |  |  |  |
| <5.7% | Ref |  | Ref |  |
| 5.7% to 6.5% | 1.01 (0.80-1.27) | 0.919 | 1.04 (0.82-1.32) | 0.766 |
| ≥6.5% | 1.05 (0.80-1.38) | 0.721 | 1.15 (0.82-1.59) | 0.418 |

Note: MCI, mild cognitive impairment; HbA1c, glycated hemoglobin; HR, hazard ratio; CI, confidence interval; Ref, reference;

Multivariate Cox regression analysis adjusted for age, gender, education, marital status, residence, annual income, smoking, heavy drinking, physical activity, CRP, hypertension, diabetes, dyslipidemia, CVD, emotional, eGFR, and abdominal obesity.

**Supplementary Table 5**. Comparison of the predictive performance of HGI and HbA1c for MCI risk.

| Variables | C-index (95%CI) | Statistic | P |
| --- | --- | --- | --- |
| HbA1c (continuous) | 0.502 (0.484-0.520) | Ref |  |
| HGI (continuous) | 0.513 (0.494-0.531) | 1.765 | 0.078 |
| HbA1c (2 categories) | 0.501 (0.494-0.508) | Ref |  |
| HbA1c (3 categories) | 0.502 (0.491-0.512) | 0.177 | 0.86 |
| HGI (5 categories) | 0.520 (0.502-0.538) | 2.138 | 0.033 |

Note: HGI, hemoglobin glycation index; HbA1c, glycated hemoglobin; MCI, mild cognitive impairment; CI, confidence interval; Ref, reference.

**Supplementary Table 6**. Characteristics of individuals based on HGI quantiles.

| Variables | Total (N=5723) | -0.368 to -0.132 (Q2) (N=1140) | <-0.368 (Q1) (N=1145) | -0.132 to 0.068 (Q3) (N=1148) | 0.068 to 0.322 (Q4) (N=1145) | ≥0.322 (Q5) (N=1145) | *P* |
| --- | --- | --- | --- | --- | --- | --- | --- |
| Age, years, Mean (±SD) | 58.13 (±8.63) | 58.05 (±8.74) | 57.75 (±8.63) | 58.09 (±8.73) | 58.17 (±8.50) | 58.61 (±8.54) | 0.197 |
| Gender, n (%) |  |  |  |  |  |  | 0.527 |
| Male | 2946 (51.48) | 598 (52.46) | 602 (52.58) | 600 (52.26) | 575 (50.22) | 571 (49.87) |  |
| Female | 2777 (48.52) | 542 (47.54) | 543 (47.42) | 548 (47.74) | 570 (49.78) | 574 (50.13) |  |
| Education, n (%) |  |  |  |  |  |  | 0.890 |
| Below high school | 4852 (84.78) | 958 (84.04) | 976 (85.24) | 968 (84.32) | 974 (85.07) | 976 (85.24) |  |
| High school and above | 871 (15.22) | 182 (15.96) | 169 (14.76) | 180 (15.68) | 171 (14.93) | 169 (14.76) |  |
| Marital status, n (%) |  |  |  |  |  |  | 0.767 |
| Married or cohabiting | 5197 (90.81) | 1028 (90.18) | 1047 (91.44) | 1045 (91.03) | 1044 (91.18) | 1033 (90.22) |  |
| Unmarried | 526 (9.19) | 112 (9.82) | 98 (8.56) | 103 (8.97) | 101 (8.82) | 112 (9.78) |  |
| Residence, n (%) |  |  |  |  |  |  | 0.492 |
| Village | 5025 (87.80) | 1001 (87.81) | 1018 (88.91) | 1002 (87.28) | 1012 (88.38) | 992 (86.64) |  |
| Town/city | 698 (12.20) | 139 (12.19) | 127 (11.09) | 146 (12.72) | 133 (11.62) | 153 (13.36) |  |
| Annual income, n (%) |  |  |  |  |  |  | 0.294 |
| <10000 | 984 (17.19) | 218 (19.12) | 192 (16.77) | 196 (17.07) | 189 (16.51) | 189 (16.51) |  |
| ≥10000 | 1354 (23.66) | 275 (24.12) | 294 (25.68) | 261 (22.74) | 254 (22.18) | 270 (23.58) |  |
| Unknown | 3385 (59.15) | 647 (56.75) | 659 (57.55) | 691 (60.19) | 702 (61.31) | 686 (59.91) |  |
| Smoking, n (%) |  |  |  |  |  |  | 0.394 |
| No current smoking | 3899 (68.13) | 753 (66.05) | 792 (69.17) | 787 (68.55) | 772 (67.42) | 795 (69.43) |  |
| Current smoking | 1824 (31.87) | 387 (33.95) | 353 (30.83) | 361 (31.45) | 373 (32.58) | 350 (30.57) |  |
| Heavy drinking, n (%) |  |  |  |  |  |  | <0.001 |
| No | 5041 (88.08) | 1000 (87.72) | 978 (85.41) | 989 (86.15) | 1031 (90.04) | 1043 (91.09) |  |
| Yes | 682 (11.92) | 140 (12.28) | 167 (14.59) | 159 (13.85) | 114 (9.96) | 102 (8.91) |  |
| Physical activity, n (%) |  |  |  |  |  |  | 0.064 |
| Insufficient | 418 (7.30) | 90 (7.89) | 72 (6.29) | 79 (6.88) | 71 (6.20) | 106 (9.26) |  |
| Sufficient | 2070 (36.17) | 400 (35.09) | 429 (37.47) | 411 (35.80) | 440 (38.43) | 390 (34.06) |  |
| Unknown | 3235 (56.53) | 650 (57.02) | 644 (56.24) | 658 (57.32) | 634 (55.37) | 649 (56.68) |  |
| Abdominal obesity, n (%) |  |  |  |  |  |  | <0.001 |
| No | 3242 (56.65) | 693 (60.79) | 657 (57.38) | 655 (57.06) | 654 (57.12) | 583 (50.92) |  |
| Yes | 2481 (43.35) | 447 (39.21) | 488 (42.62) | 493 (42.94) | 491 (42.88) | 562 (49.08) |  |
| CRP, M (Q₁, Q₃) | 1.05 (0.56, 2.14) | 0.96 (0.54, 1.95) | 1.02 (0.54, 2.18) | 1.03 (0.55, 2.03) | 1.04 (0.54, 2.07) | 1.21 (0.61, 2.41) | 0.001 |
| Hypertension, n (%) |  |  |  |  |  |  | 0.056 |
| No | 3481 (60.82) | 698 (61.23) | 667 (58.25) | 722 (62.89) | 720 (62.88) | 674 (58.86) |  |
| Yes | 2242 (39.18) | 442 (38.77) | 478 (41.75) | 426 (37.11) | 425 (37.12) | 471 (41.14) |  |
| Diabetes, n (%) |  |  |  |  |  |  | <0.001 |
| No | 4808 (84.01) | 1033 (90.61) | 831 (72.58) | 1057 (92.07) | 1056 (92.23) | 831 (72.58) |  |
| Yes | 915 (15.99) | 107 (9.39) | 314 (27.42) | 91 (7.93) | 89 (7.77) | 314 (27.42) |  |
| FBG, mg/dL, M (Q₁, Q₃) | 102.24 (94.68, 112.50) | 102.24 (95.58, 110.34) | 109.98 (100.62, 126.72) | 100.98 (94.14, 108.90) | 99.00 (92.52, 106.20) | 100.98 (91.98, 116.46) | <0.001 |
| HbA1c, %, M (Q₁, Q₃) | 5.10 (4.90, 5.40) | 4.90 (4.80, 5.10) | 4.70 (4.50, 4.90) | 5.10 (5.00, 5.20) | 5.30 (5.20, 5.40) | 5.70 (5.50, 6.20) | <0.001 |
| Dyslipidemia, n (%) |  |  |  |  |  |  | 0.009 |
| No | 1880 (32.85) | 398 (34.91) | 368 (32.14) | 382 (33.28) | 402 (35.11) | 330 (28.82) |  |
| Yes | 3843 (67.15) | 742 (65.09) | 777 (67.86) | 766 (66.72) | 743 (64.89) | 815 (71.18) |  |
| CVD, n (%) |  |  |  |  |  |  | 0.270 |
| No | 4909 (85.78) | 988 (86.67) | 997 (87.07) | 979 (85.28) | 982 (85.76) | 963 (84.10) |  |
| Yes | 814 (14.22) | 152 (13.33) | 148 (12.93) | 169 (14.72) | 163 (14.24) | 182 (15.90) |  |
| Emotional, n (%) |  |  |  |  |  |  | 0.591 |
| No | 5679 (99.23) | 1132 (99.30) | 1138 (99.39) | 1135 (98.87) | 1136 (99.21) | 1138 (99.39) |  |
| Yes | 44 (0.77) | 8 (0.70) | 7 (0.61) | 13 (1.13) | 9 (0.79) | 7 (0.61) |  |
| eGFR, n (%) |  |  |  |  |  |  | 0.038 |
| <60 | 108 (1.89) | 14 (1.23) | 32 (2.79) | 24 (2.09) | 15 (1.31) | 23 (2.01) |  |
| ≥60 | 5615 (98.11) | 1126 (98.77) | 1113 (97.21) | 1124 (97.91) | 1130 (98.69) | 1122 (97.99) |  |
| Follow time, years, Mean (±SD) | 5.44 (±1.97) | 5.48 (±1.97) | 5.48 (±1.95) | 5.42 (±2.00) | 5.50 (±1.92) | 5.34 (±2.01) | 0.335 |
| MCI, n (%) |  |  |  |  |  |  | 0.169 |
| No | 4635 (80.99) | 953 (83.60) | 921 (80.44) | 922 (80.31) | 923 (80.61) | 916 (80.00) |  |
| Yes | 1088 (19.01) | 187 (16.40) | 224 (19.56) | 226 (19.69) | 222 (19.39) | 229 (20.00) |  |
| HGI, M (Q₁, Q₃) | -0.03 (-0.29, 0.25) | -0.24 (-0.30, -0.18) | -0.57 (-0.76, -0.46) | -0.03 (-0.08, 0.02) | 0.18 (0.12, 0.25) | 0.56 (0.42, 0.86) | <0.001 |

Note: HGI, hemoglobin glycation index; CRP, C-reactive protein; FBG, fasting plasma glucose; HbA1c, glycated hemoglobin; CVD, cardiovascular diseases; eGFR, estimated glomerular filtration rate; MCI, mild cognitive impairment.
